# Impairment of the Cell Wall Ligase, LytR-CpsA-Psr Protein (LcpC), in Methicillin Resistant *Staphylococcus aureus* Reduces Its Resistance to Antibiotics and Infection in a Mouse Model of Sepsis

**DOI:** 10.3389/fmicb.2020.00557

**Published:** 2020-04-16

**Authors:** Fan Li, Dongsheng Zhai, Zhaowei Wu, Yan Zhao, Dandan Qiao, Xin Zhao

**Affiliations:** ^1^College of Animal Science and Technology, Northwest A&F University, Yangling, China; ^2^Department of Pharmacology, School of Pharmacy, Fourth Military Medical University, Xi’an, China; ^3^School of Physical Science and Technology, ShanghaiTech University, Shanghai, China; ^4^Department of Animal Science, McGill University, Montreal, QC, Canada

**Keywords:** *Staphylococcus aureus*, MRSA, LcpC, β-lactams, glycopeptides, adhesion, pathogenicity

## Abstract

*Staphylococcus aureus* is a major opportunistic pathogen, infecting animals, and human beings. The bacterial cell wall plays a crucial role in antimicrobial resistance and its infection to host cells. Peptidoglycans (PGs) are a major component of the cell wall in *S. aureus*, which is heavily decorated with wall teichoic acids (WTAs) and capsular polysaccharides (CPs). The ligation of WTAs and CPs to PGs is catalyzed by LytR-CpsA-Psr (LCP) family proteins, including LcpA, LcpB, and LcpC. However, the involvement of LcpC in antimicrobial resistance of *S. aureus* and its infection to host cells remains unknown. By creating the LcpC-knockout strains, we showed that the deficiency in LcpC decreased the antimicrobial resistance to β-lactams and glycopeptides and impeded the binding to various epithelial cells. These changes were accompanied by the morphological changes in bacterial cell wall. More importantly, the knockout of LcpC significantly reduced the pathogenicity of methicillin-resistant *S. aureus* (MRSA) in mice. Our results suggest that LcpC might be an appealing target for developing a therapeutic approach against MRSA infections.

## Introduction

*Staphylococcus aureus* can cause both superficial and invasive infections in humans and animals. *S. aureus* sometimes turns into an extremely threatening pathogen when it traverses the epithelial barrier and gains access to internal tissues from where it can infect most organs and cause a broad spectrum of diseases, including pneumonia, endocarditis, and staphylococcal scalded skin syndrome (Peacock and Paterson, 2015). *S. aureus* is also a major pathogen for mastitis in dairy cows and ulcerative pododermatitis in poultry (Peacock and Paterson, 2015). Over the years, continued selective pressure by overuse or misuse of different antimicrobials has resulted in microorganisms with multi-drug resistance (Alekshun and Levy, 2007). The emergence of multi-drug-resistant *S. aureus* and its internalization into host cells further complicate the treatment of its infection (Lowy, 1998).

Multi-drug-resistant *S. aureus* poses a significant threat to public healthcare and animal production. In particular, the emergence of methicillin-resistant *S. aureus* (MRSA) narrowed the therapeutic options (Peacock and Paterson, 2015). Most MRSA strains harbor a *mecA* gene, which encodes penicillin-binding protein 2a (PBP2a) with a significantly reduced affinity to β-lactams. PBP2a confers MRSA, a pan-resistance to most β-lactam antibiotics (Peacock and Paterson, 2015). Therefore, the glycopeptide antibiotics, such as vancomycin and teicoplanin, have been used as the last resort to treat MRSA (David and Daum, 2017). To date, vancomycin-resistant strains are still rare (Mcguinness et al., 2017). However, *S. aureus* with an intermediate-level vancomycin resistance (VISA) have been frequently identified in hospitals (Adams et al., 2017), communities (Abdel-Maksoud et al., 2016), and farms (Bhattacharyya et al., 2016). In addition to antimicrobial resistance, *S. aureus* is capable of evading antimicrobial chemotherapy and host immune defense via internalization into host cells (Alexander and Hudson, 2001). The adhesion to host cells, as the first step of internalization, is facilitated by adhesins (Foster et al., 2014). Both antimicrobial resistance and the adhesion ability of *S. aureus* are significantly affected by the outer cell wall architecture (Rajagopal and Walker, 2017). In gram-positive bacteria, peptidoglycans (PGs) are a major component of the bacterial cell wall, which are heavily decorated by a series of glycopolymers, including the wall teichoic acids (WTAs), and the capsular polysaccharides (CPs) (Rajagopal and Walker, 2017). These glycopolymers also contribute to adherence of *S. aureus* to host cells (Rajagopal and Walker, 2017). The polymers of WTAs and CPs are transported to the external surface of the bacterial membrane, and can be subsequently attached to PGs by LytR-CpsA-Psr (LCP) proteins (Chan et al., 2014; Schaefer et al., 2017).

LCP proteins are present in most Gram-positive microorganisms, and are variously essential for normal septum formation during cell division (Johnsborg and Havarstein, 2009), correct cell division, cell autolysis, biofilm formation (Chatfield et al., 2005), the tolerance to acid, and oxidative stress (Wen et al., 2006). *S. aureus* contains three *lcp* genes, termed *lcpA*, *lcpB*, and *lcpC* (Chan et al., 2013). Complete loss of WTA occurred when all three *lcp* genes were deleted, significantly reducing the antimicrobial resistance (Dengler et al., 2012) and impairing the cell division process (Chan et al., 2013). The deficiency of LcpA reduced the resistance to oxacillin (Schaefer et al., 2017) in heterogeneous vancomycin-intermediate *S. aureus* (hVISA) strain Mu3 (Katayama et al., 2016).

It has been proven that LcpC attaches CPs to PGs (Schaefer et al., 2017; Rausch et al., 2019). Despite the biological importance of LcpC as a ligase, two important questions still remain: whether and how LcpC could influence the antimicrobial resistance of *S. aureus* and whether and how the changes of the deficiency in LcpC could impair the pathogenicity of *S. aureus* to the host. Here, we deleted *lcpC* in *S. aureus* strains and compared the multiple antibiotic resistance, as well as the adherence of *S. aureus* to host cells among the wild types, mutants, and complements. In addition, electron microscopic analyses were performed to observe the cell wall morphology, and expression of genes involved in cell wall biosynthesis was investigated. Finally, an MRSA-induced septic shock mouse model was used to determine the roles of LcpC in host–pathogen interaction. Our results clearly demonstrate that LcpC in *S. aureus* plays a crucial role in antimicrobial resistance and the infection to host cells.

## Materials and Methods

### Bacterial Strains, Plasmids, and Growth Conditions

The bacterial strains and plasmids used in this study are listed in Supplementary Table S1. *Escherichia coli* DH5α were grown in the LB medium with appropriate antibiotics. *S. aureus* and its derivative strains were grown in the tryptic soy broth (TSB) medium supplemented with chloramphenicol when necessary. The Mueller-Hinton agar supplemented with 2% NaCl was used to test the susceptibilities of *S. aureus* to antibiotics.

### Construction of the LcpC Mutant and Its Complement

Oligonucleotides used in this study are listed in Supplementary Table S2. The deletions of *lcpC* were performed as previously described (Bae and Schneewind, 2006). Briefly, to construct the *lcpC* mutants (Δ*lcpC*), a fragment containing 900-bp upstream and 900-bp downstream of *lcpC* was inserted into pKZ2 via Gibson assembly, yielding pKZ2-Δ*lcpC*. Then, the plasmid pKZ2-Δ*lcpC* was electroporated into *S. aureus*. By shifting temperature to 43°C, the pKZ2-Δ*lcpC* was integrated into the chromosome. Then, the culture was transferred to 30°C overnight for loss of plasmids. Subsequently, the culture was 10^4^-fold diluted and 100-μl aliquots were spread on TSA containing 0.5 μg/ml of anhydrotetracycline (ATc) and incubated at 37°C for overnight. PCRs were carried out to screen the colonies with the desired deletion.

To complement the *lcpC* mutation *in situ*, a complete native expression cassette of *lcpC*, the upstream (900 bp), and the downstream (900 bp), were inserted into pKZ2. Moreover, an *Xho*1 restriction site was inserted after the stop codon of *lcpC* for distinguishing the complements from the wild type, yielding pKZ2-*lcpC*-*Xho*1. The knock-in step was similar to the deletion.

### Antibiotic Susceptibility Testing of Δ*lcpC*

Spot assay was performed to determine the susceptibilities of Δ*lcpC* strains (Eyraud et al., 2014). Briefly, overnight cultures were diluted to 0.5-McFarland standard. Then, five consecutive 10-fold dilutions were prepared. One microliter of each dilution was spotted on the Mueller–Hinton agar with 2% NaCl supplemented with oxacillin, teicoplanin, cefazolin, penicillin, and vancomycin, respectively. The plates were incubated for 24–36 h at 35°C.

In parallel, the growth curves of *S. aureus* and their derivative strains were also recorded in the TSB medium supplemented with antibiotics, in order to compare the susceptibilities of the *lcpC* mutants to the wild types and the complements. Overnight cultures were inoculated to fresh TSB medium with appropriate antibiotics at a ratio of 1:100. The cultures were incubated in a shaker at 37°C and the OD_600_ values were determined at a 1-h interval for 8 h.

### Adhesion of *S. aureus* to Host Cells

A549 cells (human lung adenocarcinoma cells, ATCC CCL-185) and MCF-7 cells (human breast cancer cells, ATCC HTB-22) were obtained from ATCC. HCMEC (human cardiac microvascular endothelial cells, ZY-603) and HaCaT (human skin keratinocytes, ZY-504) were obtained from Shanghai ZeYe Biotech Co., Ltd., Cell lines were extensively used in investigations of *S. aureus* adhesion. These cells were maintained in the DMEM (HyClone) supplemented with 10% fetal bovine serum (FBS) (Biological Industries). The adherence of *S. aureus* to epithelial cells was first determined by counting the CFUs of adhered bacteria as previously described (Yang and Ji, 2014). Briefly, each well of confluent monolayers cells (approximately 10^6^ cells) in 24-well plate was washed by PBS and approximately 10^8^ CFU of bacteria suspended in 1-ml DMEM supplemented with 10% FBS was added into each well. After 1-h incubation at 37°C in a 5% CO_2_ incubator, cells of each well were washed by PBS to remove non-adherent bacteria. Then, adhered bacteria were released by the treatment with 0.25% trypsin-EDTA and 0.25% Triton X-100. The mixture was diluted and plated on TSA plates in triplicate. The colonies were counted after incubating overnight at 37°C. The adherence was calculated by dividing the original CFU of inoculum by the adhered CFU. All experiments were performed three times.

In parallel, the adherence of *S. aureus* to epithelial cells was also determined by flow cytometry. A constitutive GFP-expressing plasmid, pSB2019, was transformed into the *S. aureus* strains as a fluorescence label (Qazi et al., 2001). In total, 10^6^ mammalian cells were collected and suspended in 1-ml DMEM supplemented with 10% FBS, then approximately 10^9^ CFU of GFP-expressed bacteria were added. After 1-h incubation at 37°C, the mixture was subjected to flow cytometry using a CytoFLEX S cytometer. Cells adhered with GFP-labeled bacteria were excited at 488 nm and the GFP fluorescence signals were detected by a 525/40 bandpass emission filter. For each sample, 10,000 events were recorded, and the cell populations and the mean fluorescence intensity (MFI) of each sample were analyzed using the CytExpert 2.3 software. All experiments were performed five times.

### Transmission Electron Microscopic Analysis

For transmission electron microscopy (TEM), the bacteria were grown in the TSB medium at 37°C with shaking. The OD_600_ of the culture was measured. Fifty milliliters of the culture in the logarithmic (OD_600_ ≈ 0.6) phase or 1 ml of the culture in the stationary phase (OD_600_ ≈ 10) was pelleted and fixed with 1-ml 2.5% glutaraldehyde and 4% paraformaldehyde in a phosphate buffer for 2 h at 4°C. The fixation reagents were washed two to three times with 0.1 M PBS. After that, the samples were second-fixed with 1% OsO4 buffer for 2 h and washed two to three times with 0.1 M PBS. The samples were dehydrated with ethanol and then immersed in the mixture of propylene oxide and Eponate 812 for 1 h. Sections of 90–100 nm were prepared, stained with uranyl acetate and lead citrate, and observed under a JEOL 1200 EX transmission electron microscope (Hitachi H-600/H-700).

### Scanning Electron Microscopic Analysis

Scanning electron microscopic (SEM) analysis were performed as described previously (Si et al., 2018). *S. aureus* were grown in TSB to stationary phase and were collected via centrifugation. Then, the pellet was washed with PBS twice and the sample was fixed with 2.5% glutaraldehyde and 4% paraformaldehyde in phosphate buffer for 6 h at 4°C. The bacterial cells were dehydrated by washing with a series of ethanol solutions (10–100%). After drying the sample, the bacterial cells were then observed with a field-emission scanning electron microscope (FE-SEM, FEI Inspect F50).

### Animal Preparation

Eighty-four 8- to 10-week old C57BL/6 mice were purchased from the Experimental Animal Center of the Fourth Military Medical University 1 week before this study. Mice were kept under specific pathogen-free conditions in 500 × 300 × 150 mm cages and given free access to water and mouse maintenance food (Jiangsu Xietong Pharmaceutical Bio-engineering Co., Ltd). The mouse room was kept at 12-h light/dark cycles with lights on at 08:00 and off at 20:00, temperature of 22°C ± 2°C, humidity of 55% ± 5%. During housing, animals were monitored twice daily for health status. No adverse events were observed. At the start of the experiment, the average weight of animals was 19.93 ± 1.51 g (mean ± SD).

### MRSA-Induced Septic Shock Mouse Model

In this animal septic shock model, a clinical MRSA strain, Mu50, and its Δ*lcpC* derivatives were used and the assay was performed as described previously (Jo et al., 2011). Briefly, mice were injected with 10^9^CFU of *S. aureus* intraperitoneally (IP). Mice were treated with oxacillin (400 mg/kg) intraperitoneally 15 min after the infection of *S. aureus*. The same oxacillin treatment was repeated three times with a 2-h interval. The mortality of mice was observed for 3 days after the infection. After 3 days, the organs including liver, spleen, and lung of one randomly selected survival mouse were collected for histological observation. Moreover, these organs in a parallel group were collected after 12-h infection, which were then homogenized and serially diluted in PBS. The mixture was plated on TSA plates and incubated at 37°C overnight for CFU counting. Furthermore, the peripheral blood of mice after 24-h infection was also collected for determining the cytokine levels of TNF-α, IL-1β, and IL-6 via ELISA Kit (Dakewe, Shenzhen, China). Once experiments were finalized, animals were sacrificed by euthanasia by four times the anesthetic doses of ketamine/xylazine combinations that were administered intraperitoneally.

### Cytokine Detection in Macrophage Cell Line

To confirm the results of the cytokine levels from the mouse model, a mouse macrophage cell line, RAW 264.7 (American Type Culture Collection, ATCC TIB-71), was used for infection with *S. aureus* as previously described (Zhai et al., 2017). Briefly, approximately 10^5^ cells were infected with 10^7^-CFU Mu50, Δ*lcpC* mutant, and the complement, respectively. After 4-h treatment, the culture supernatants were collected for determining the levels of three cytokines (TNF-α, IL-1β, and IL-6). To determine transcriptional levels of three cytokines, total RNA was extracted with Trizol reagent (Life Technologies, United States) and reversely transcribed with M-MuLV reverse transcriptase (Takara, Japan). Real-time PCR was performed with universal SYBR Green PCR Mix (Takara, Japan) on BioRad CFX96 Real-Time PCR Detection System (BioRad, United States) by primers in Supplementary Table S2. Furthermore, the protein levels of cytokines in RAW 264.7 were also determined by ELISA Kit (Dakewe, Shenzhen, China) according to the manufacturer’s instructions.

## Results

### LcpC Contributes to Antimicrobial Resistance in *S. aureus*

Even though the deficiency of LcpA reduced the resistance to oxacillin (Schaefer et al., 2017), the function of LcpC in antimicrobial resistance in *S. aureus* is still unknown. To investigate the role of *lcpC* in antimicrobial resistance of *S. aureus*, *lcpC* knockout mutants were created by allelic exchange in four *S. aureus* strains, including two MRSA strains, Mu50 (a hospital-associated MRSA) and BA01611 (a livestock-associated MRSA) (Kuroda et al., 2001; Wu et al., 2015), and two MSSA strains, Newman (a hospital-associated MSSA) and RN4220 (a laboratory strain) (Baba et al., 2008; Nair et al., 2011). The susceptibilities to cell wall active antibiotics β-lactams (oxacillin, cefazolin, penicillin) and glycopeptides (teicoplanin, vancomycin), as well as antibiotics in other classes (ciprofloxacin, tetracycline, chloramphenicol, and streptomycin) were determined in the wild-type strains, the *lcpC* knockout mutants, and the *in situ* complement strains by spot assays. The growth curves in the TSB medium supplemented with antibiotics were also used to corroborate the results from the spot assay.

**FIGURE 1:**
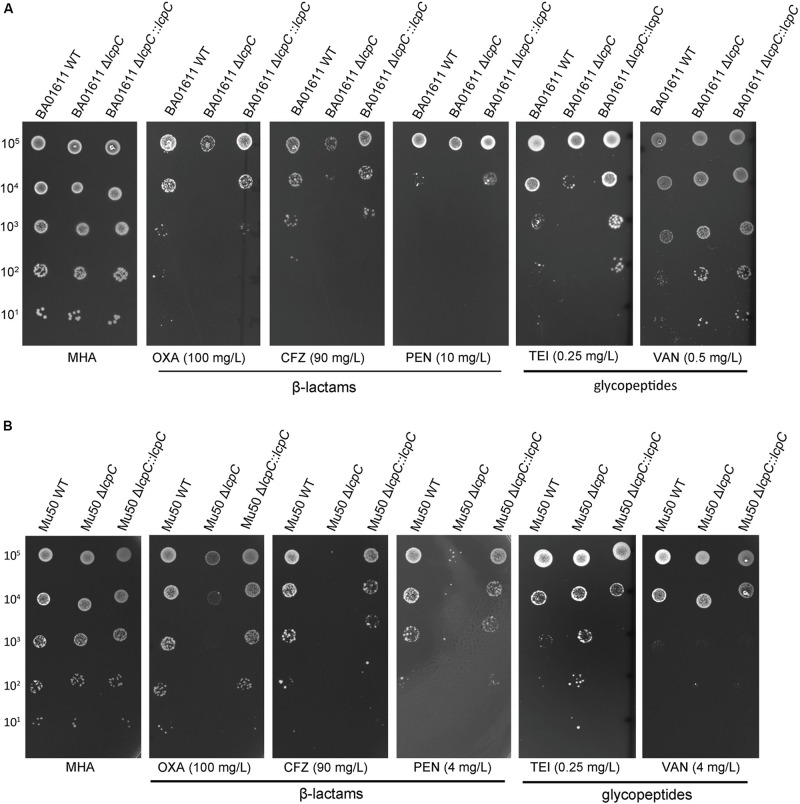
β-Lactam and glycopeptide susceptibility testing by spot assay for MRSA BA01611 and MRSA Mu50. Serial dilutions of *S. aureus* were spotted on the MHA + 2% NaCl medium and then incubated for 24–36 h at 35°C. The first 1-μl spot dilution corresponds to 10^5^ CFU. **(A)** MRSA BA01611 cultured on the MHA + 2% NaCl medium with no antibiotic (MHA), 100 mg/L oxacillin, 90 mg/L cefazolin, 10 mg/L penicillin, 0.25 mg/L teicoplanin, and 0.5 mg/L vancomycin. **(B)** MRSA Mu50 cultured on the MHA + 2% NaCl medium with no antibiotic (MHA), 100 mg/L oxacillin, 90 mg/L cefazolin, 4 mg/L penicillin, 0.25 mg/L teicoplanin, and 4 mg/L vancomycin.

**FIGURE 2:**
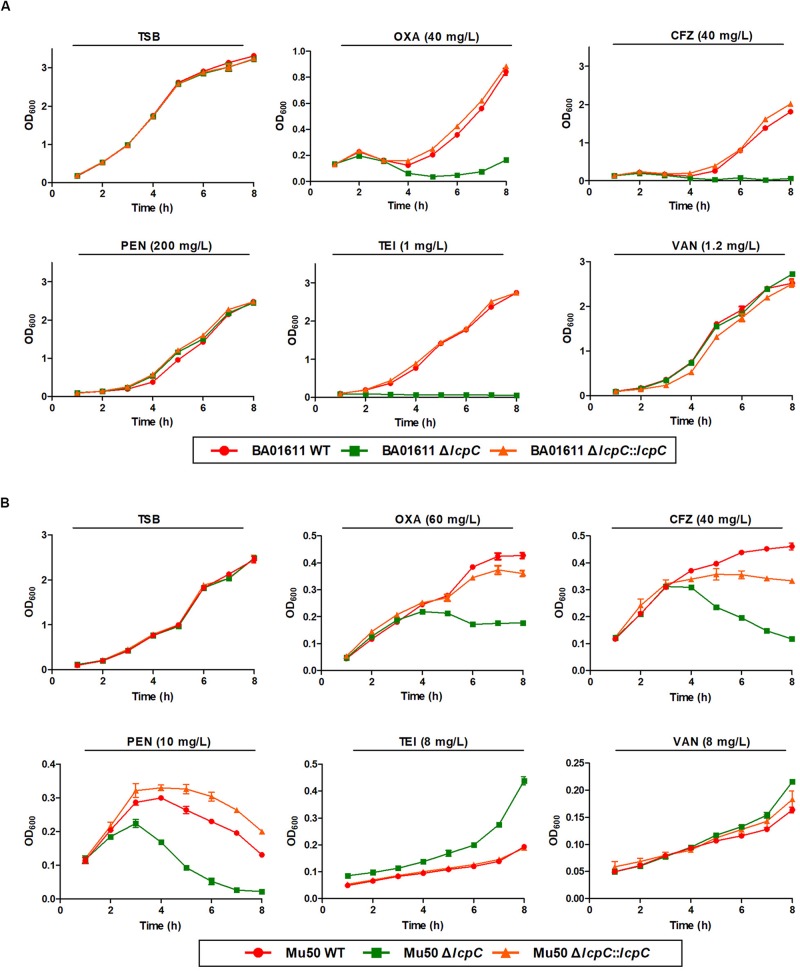
β-Lactam and glycopeptide susceptibility assay by growth curve for MRSA BA01611 and Mu50. The growth assay of the wild-type, the Δ*lcpC*, and the *lcpC* complementary strains in the TSB medium was carried out at 37°C. **(A)** MRSA BA01611 in the TSB medium with no antibiotic, 40 mg/L oxacillin, 40 mg/L cefazolin, 200 mg/L penicillin, 1 mg/L teicoplanin, and 1.2 mg/L vancomycin. **(B)** MRSA Mu50 in the TSB medium with no antibiotic, 60 mg/L oxacillin, 40 mg/L cefazolin, 10 mg/L penicillin, 8 mg/L teicoplanin, and 8 mg/L vancomycin. Data are represented as the means ± S.D. (error bars) of the results of three independent experiments.

As shown in Figure 1, deficiency of LcpC significantly reduced the resistance to β-lactam antibiotics including oxacillin, cefazolin, and penicillin in the MRSA strains. In contrast, the resistance to other antibiotics including ciprofloxacin, tetracycline, chloramphenicol, streptomycin, and vancomycin was not significantly affected by the deficiency of LcpC in MRSA strains (Supplementary Figure S1). Intriguingly, the knockout of *lcpC* affected the resistance to teicoplanin in different manners in the two MRSA strains. The resistance to teicoplanin was reduced by the *lcpC* knockout in BA01611 (Figures 1A, 2A), while the deficiency of LcpC increased the resistance to teicoplanin in Mu50 (Figures 1B, 2B). On the other hand, the resistance to oxacillin and cefazolin was decreased in Newman by the knockout of *lcpC* (Supplementary Figure S2A). Additionally, deficiency of LcpC reduced the resistance to cefazolin and vancomycin in RN4220 (Supplementary Figure S2B). These results indicated that LcpC contributed to the resistance to cell wall active antibiotics in both MRSA and MSSA strains.

### Deficiency in LcpC Impaired the Adhesion of *S. aureus* to Host Cells

In order to study the possible involvement of LcpC in the adhesion of *S. aureus* to host cells, A549 (human pulmonary epithelial cell line), MCF7 (human mammary gland epithelial cell line), HCMEC (human cardiac microvascular endothelial cells), and HaCaT (adult skin keratinocytes) were used in our study. These four cell lines are frequently used as *in vitro* models to investigate bacterial infectious diseases, including pneumonia (Nguyen et al., 2014), mastitis (Hickey et al., 2018), endocarditis (Ferrero et al., 2011), and staphylococcal scalded skin syndrome (Edwards et al., 2011).

**FIGURE 3:**
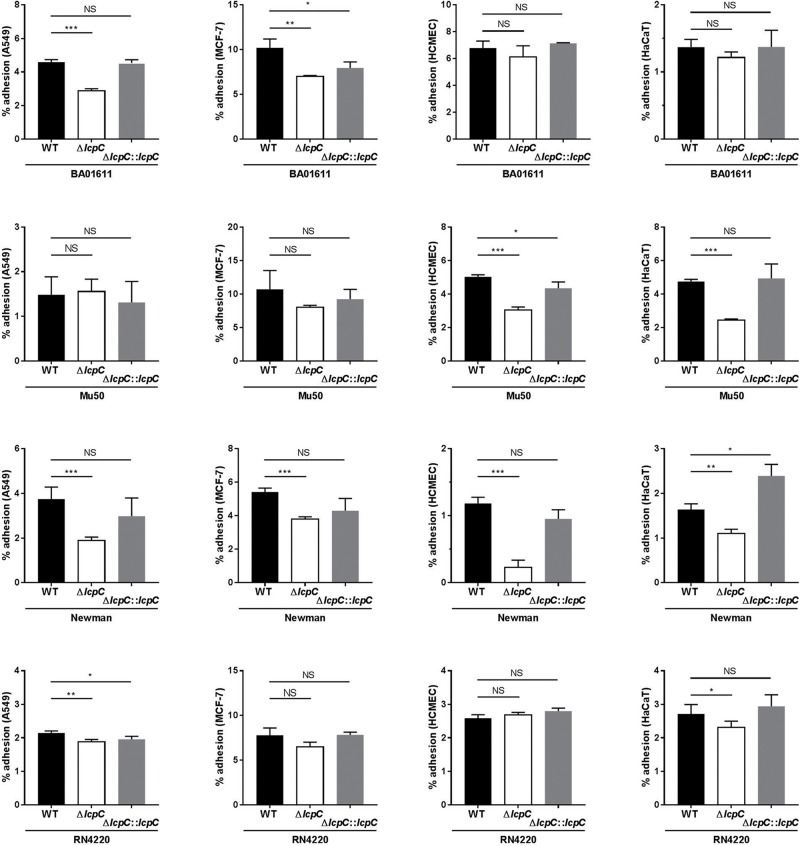
The percent adherence of *S. aureus* to epithelial cells by colony counting on TSA plates. The adhesion between *S. aureus* strains (wild type, knockout type and complement type of BA01611, Mu50, Newman and RN4220) and four cells lines (A549, MCF-7, HCMEC and HaCaT) was analyzed by counting the colonies on TSA plate. After 1-h incubation with each strain, 10^6^ cells with adherent bacteria from each well were diluted and then plated on TSA. The percent adherence was calculated as 100 × (CFU of adherent bacteria/CFU of inoculated bacteria). Two-tailed unpaired Student’s *t* test was performed. NS, not significant, *P < 0.05, **P < 0.01, or ***P < 0.001. Data are represented as the means ± S.D. (error bars) of the results of three independent experiments.

The adherence of *S. aureus* to the host cells was first determined by CFU counting of adhered bacteria. As shown in Figure 3, adherence of each Δ*lcpC* was significantly impaired in at least two types of cell lines. For Newman strain, adherence of its Δ*lcpC* strain was significantly reduced in all four cell lines (Figure 3). Various *S. aureus* strains exhibited differed adherent preferences. Moreover, the adhesion of *S. aureus* to the MCF-7 cell line was significantly higher than those to the other three cell lines (Figure 3).

**FIGURE 4:**
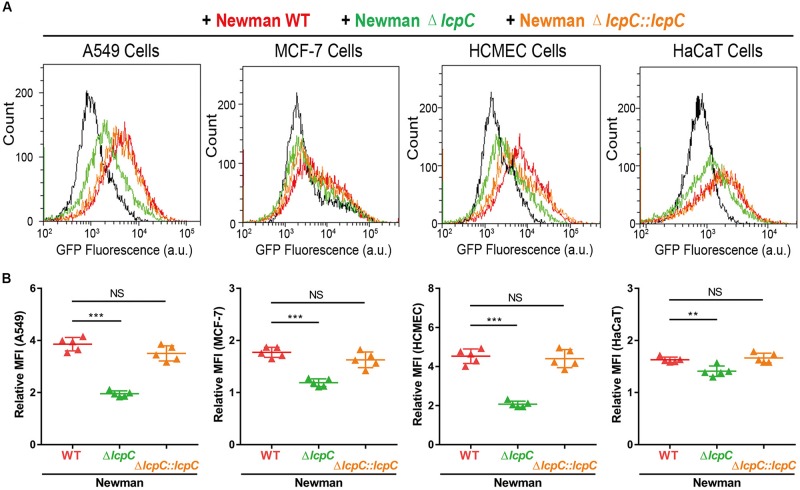
Adhesion of *S. aureus* Newman to cell lines by flow cytometry. The adhesion between GFP-containing Newman strains (Newman WT, Newman Δ*lcpC*, and Newman Δ*lcpC:lcpC*) and four cell lines (A549, MCF-7, HCMEC, and HaCaT) were analyzed by flow cytometry at 1-h post adhesion. Ten thousand cells were collected and GFP fluorescence intensity was calculated by CytoFLEX S (Beckman Coulter). **(A)** The GFP fluorescence distribution of cell populations. Red, green, and orange represent the epithelial cells adhered by Newman WT, Newman Δ*lcpC*, and Newman Δ*lcpC*:*lcpC*, respectively. Cell lines without *S. aureus* were examined as the control. **(B)** The mean fluorescence intensity (MFI) of each sample harboring specific cells. Relative MFI = MFI of epithelial cells adhered by *S. aureus* strains/MFI of control cells. Two-tailed unpaired Student’s *t* test was performed. NS, not significant, **P < 0.01, ***P < 0.001. *n* = 5 replicates.

To corroborate the results obtained by the CFU counting, flow cytometry assays were performed. In this assay, *S. aureus* strains were labeled by a constitutively GFP-expressing plasmid and then used to infect the suspended host cells with a multiplicity of infection (MOI) at 10^3^–10^4^ for 1 h. As shown in Figure 4 and Supplementary Figure S3, the *lcpC* mutants of BA01611 and Newman exhibited reduced adherence to all four cell lines. Moreover, the decreased adherence in Mu50 Δ*lcpC* was observed in three cell lines, including MCF-7, HCMEC, and HaCaT. The changes in adherence for RN4220 and its derivative strains were consistent in two methods. In contrast to the results from CFU counting on Mu50 and its derivative strains, a significant alteration in the adherence on MCF-7 cell line was only observed by the flow cytometry, suggesting that the flow cytometry assay was more sensitive than the CFU counting method.

Collectively, our experiment demonstrated that LcpC played a crucial role in the adherence of both MRSA and MSSA to the host cells. Their adherence to host cells was all impaired when *lcpC* was deleted.

### Deletion of *LcpC* Affected the Cell Wall Architecture in *S. aureus*

**FIGURE 5:**
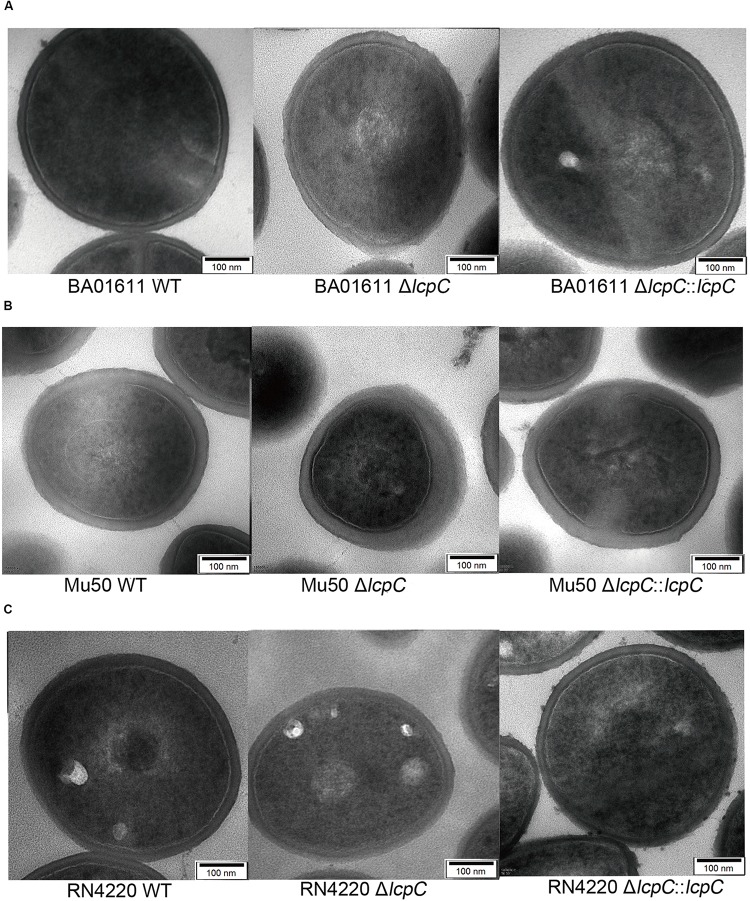
TEM analysis of *S. aureus* at the stationary phase. Analysis of cell morphology of *S. aureus* cultured in the TSB medium overnight and 1 ml of stationary phase culture was collected for TEM assay. From left to right: **(A)** BA01611 WT and its derivatives. **(B)** Mu50 WT and its derivatives. **(C)** RN4220 WT and its derivatives.

Whether deletion of *lcpC* alone can alter staphylococcal morphology remains unknown. In order to investigate whether the alterations in antimicrobial resistance and adherence to host cells were induced by changes in the staphylococcal outer cell wall architectures, TEM and scanning electron microscopy (SEM) were used to observe the cell wall architecture in these *S. aureus* strains. As shown in Figure 5 (the stationary phase) and Supplementary Figure S4 (the logarithmic phase), the cell wall architecture in these *lcpC* mutants (BA01611, Mu50, and RN4220) was significantly altered to be rougher and fuzzier, but those in the wild types and the complement strains are intact and smooth.

**FIGURE 6:**
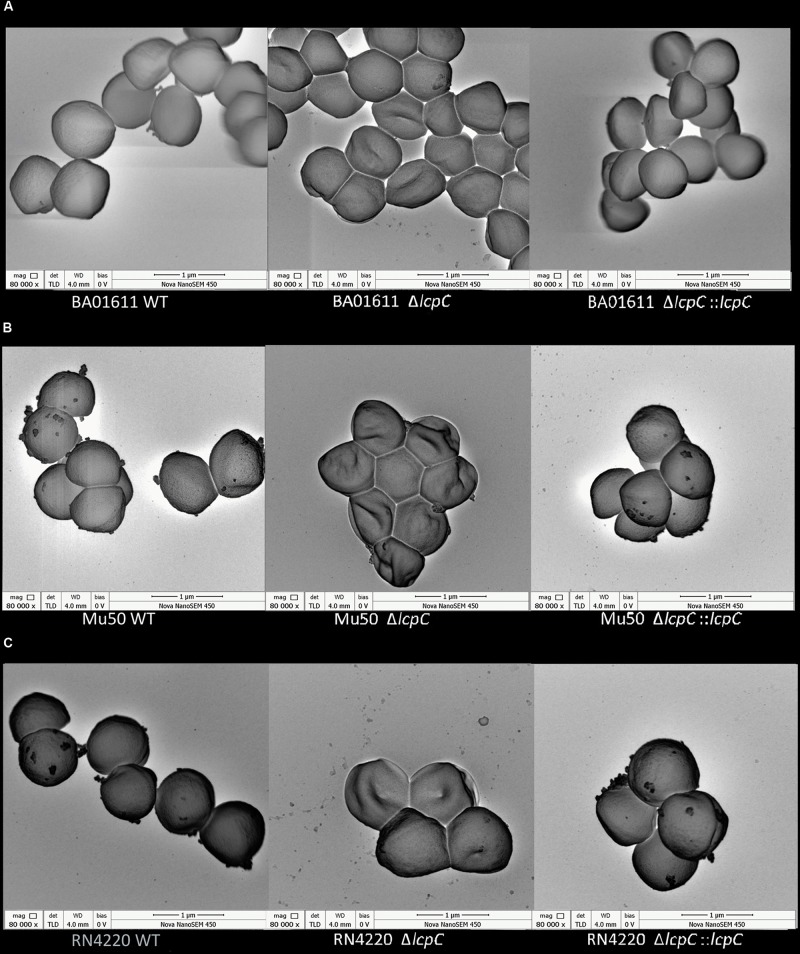
SEM analysis of *S. aureus*. Analysis of cell morphology of *S. aureus* cultured in the TSB medium overnight and 1 ml of stationary phase culture was collected for SEM assay. From left to right: **(A)** BA01611 WT and its derivatives. **(B)** Mu50 WT and its derivatives. **(C)** RN4220 WT and its derivatives.

Moreover, SEMs showed that the absence of LcpC caused dents and hollows on surfaces. The mutants seemed to be deflated. In contrast, the surfaces of wild-type and complement strains maintained a typical grape-like staphylococcal shape (Figure 6). However, these changes of the cell wall were not obviously observed by electron microscopy in Newman strains (Supplementary Figure S5).

### Δ*lcpC* Attenuated Pathogenicity in a Mouse Septic Shock Model

**FIGURE 7:**
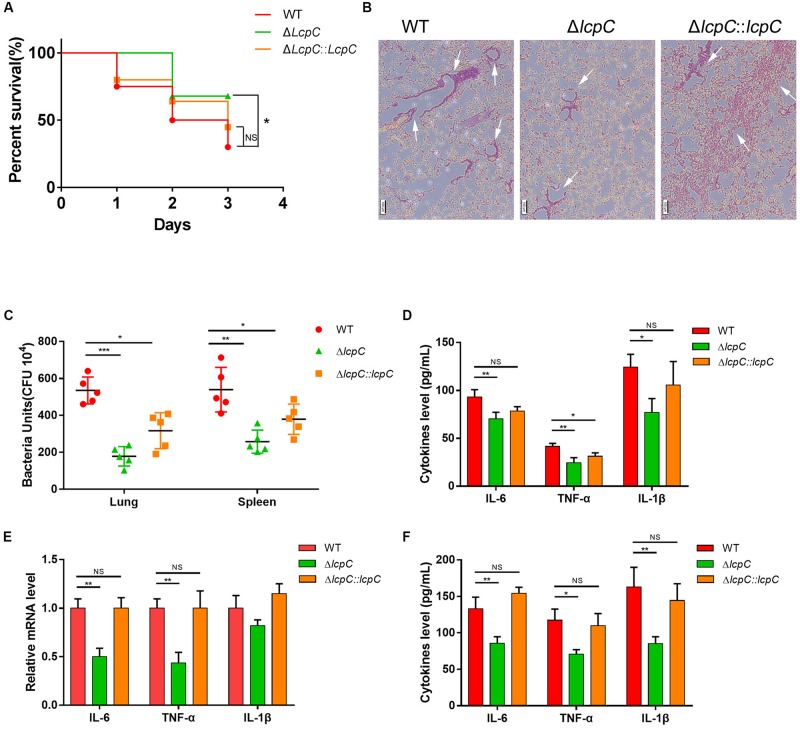
Lcpc in the mouse sepsis model. Mice were injected intraperitoneally with live 1 × 10^9^ CFU MRSA Mu50 (WT, Δ*lcpC* and the complement) and then treated with oxacillin intraperitoneally after infection. **(A)** The survival rate of mice infected with MRSA Mu50 (WT, Δ*lcpC* and the complement) within 3 days (*N* = 20/group) and was analyzed by log-rank test. NS, not significant, *P < 0.05. **(B)** Representative sections of mice infected with MRSA strains (Mu50 WT, Mu50 Δ*lcpC*, and Mu50 Δ*lcpC*: *lcpC*) for 24 h were stained with hematoxylin and eosin (HE) Scale bar, 100 μm. Infiltration of immune cells was presented with white arrows. **(C)** Colony-forming units (c.f.u.) from spleen and lung after infection with MRSA 12 h. **(D)** Levels of cytokines (IL-10, IL-6, TNF-α, and IL-10) in blood after infection for 24 h. *n* = 3 replicates **(E)** Relative mRNA expression of the inflammatory cytokine level (IL-1β, IL-6, and TNF-α) in RAW264.7 cells co-culture with Mu50 strains for 4 h. *n* = 3 replicates. **(F)** Cytokine (IL-1β, IL-6, and TNF-α) levels in cellular supernatant from RAW264.7 cells co-culture with Mu50 strains for 4 h. *n* = 3 replicates. Two-tailed unpaired Student’s *t* test was performed. NS, not significant, *P < 0.05, **P < 0.01, ***P < 0.001. *n* = 3 replicates.

The decline in the antimicrobial resistance and the adherence to host cells led to a hypothesis that the deficiency in LcpC may reduce the toxicity of *S. aureus in vivo*. Thus, a mouse septic shock model with an MRSA was used. Mice were intraperitoneally infected with the clinical MRSA Mu50 and its derivative strains as described by Kuroda et al. (2001). The mice were then treated with 400 mg/kg oxacillin three times. The survival rates within 3 days were recorded to be 70% in the Δ*lcpC*. In contrast, the wild-type and complement group maintained the survival rates at 30% and 50%, respectively (Figure 7A). The histopathology in lung tissue by hematoxylin and eosin staining revealed that the Δ*lcpC* caused lower inflammation than those of the wild-type and the complement strains (Figure 7B). Moreover, less bacteria were invaded in the lung and spleen in the Δ*lcpC* than in the wild-type and the complement strains (Figure 7C). However, no significant change was observed in the liver and kidney (data not shown). In addition, levels of IL-6, TNF-α, and IL-1β were significantly lower in the Δ*lcpC* (Figure 7D).

The cytokine changes were also confirmed *in vitro* through co-culturing the mouse macrophage cell line RAW 264.7 with the MRSA Mu50 and its derivative strains for 4 h. The transcript levels of IL-6 and TNF-α (Figure 7E) and protein levels of IL-6, TNF-α, and IL-1β (Figure 7F) were significantly reduced when infected with the Δ*lcpC*. These results clearly showed that LcpC contributes to the pathogenicity of *S. aureus*.

## Discussion

The results from this study clearly show that LcpC is very important for the antimicrobial resistance and the adhesion of *S. aureus* to host cells. It is well known that WTAs and CPs, which are attached to PGs in cell walls by LCP family proteins, have essential roles in infections caused by major human pathogens, such as *S. aureus*, *S. pneumoniae, Bacillus anthracis*, and *Mycobacterium tuberculosis* (Weidenmaier and Peschel, 2008). However, the role and contribution of LCP family proteins to antimicrobial resistance and the pathogenicity to hosts in Gram-positive pathogens such as *S. aureus* are much less clear. In this study, we have focused on LcpC, one of three LCP family members, since it was reported that *sacol2302* (*lcpC*) in MRSA strain COL adapted to high levels of oxacillin was overexpressed in comparison with the parent COL strain (Solis et al., 2014).

Antimicrobial resistance presents an important hurdle in the therapy of staphylococcal infections. Additionally, bacterial adhesion and then the invasion of host cells exacerbate the difficulty to cure the infections. Thus, there is a pressing need to develop new and more effective treatment strategies. In this study, we found that the absence of LcpC might both relieve these two situations. This study demonstrated that the absence of LcpC decreased the resistance to β-lactams in MRSA (BA01611 and Mu50) and to certain glycopeptide and β-lactam antibiotics in MSSA (Newman and RN4220). The change of the resistance could be introduced by changes of outer cell wall architectures. Both TEM and SEM results showed that *lcpC* mutants had abnormal cell shape, as well as the dented and hollowed cell envelope, which may allow the cell wall active antibiotics easier access to the peptidoglycan terminus, therefore reducing the antimicrobial resistance to β-lactam and certain glycopeptide antibiotics. The abnormal cell shape might be caused by impairing cell wall biosynthesis, since LcpC was capable of transferring WTA substrates to PG oligomers (Schaefer et al., 2017). It has been reported that the deficiency of WTA due to mutation of the *tar* gene cluster sensitized MRSA strains to β-lactams (Campbell et al., 2011; Brown et al., 2012). Interestingly, the absence of *lcpC* affected the resistance to teicoplanin in different manners in the two MRSA strains, increasing the resistance to teicoplanin in Mu50 and decreasing the resistance to teicoplanin in BA01611. The differences of these two MRSA strains might be due to the different resistance to glycopeptide of their parental strains. Mu50 is a vancomycin-intermediate *S. aureus* (VISA), while BA01611 is a vancomycin-sensitive *S. aureus*. Both Mu50 and Mu3 contain a mutation in another LCP member, *lcpA*, and both are VISA (Katayama et al., 2016). Introduction of mutated *lcpA* into vancomycin-susceptible *S. aureus* (VSSA) strain N315ΔIP increased the level of VISA (Katayama et al., 2016). Thus, it seems that both normal LcpA and LcpC proteins could keep VISA strains sensitive to teicoplanin.

This study was also the first one to report that the deficiency of LcpC impaired the adherence to host cells in both MRSA and MSSA strains. These changes could be induced by impairment of both WTA and CP biosynthesis in cell wall, because LcpC is also the cell wall ligase of CP to PG (Rausch et al., 2019). *S. aureus* WTAs and CPs are both required for bacterial adhesion to host tissue (O’riordan and Lee, 2004; Weidenmaier et al., 2004). Additionally, all strains used in this study were encapsulated (Luong and Lee, 2002; Jansen et al., 2013), including BA01611 (unpublished).

The most important finding from this study was the demonstration that the Δ*lcpC* caused the attenuation in pathogenicity in a mouse septic shock model with oxacillin treatments. In comparison with the wild type, the *lcpC*-mutant reduced mortality, decreased invasive bacteria in spleen and lung, and reduced levels of cytokine IL-6, TNF-α, and IL-1β. These *in vivo* results were fully supported by our *in vitro* results with Mu50 Δ*lcpC*. It suggests that the presence of LcpC not only facilitates murine *S. aureus* carriage but also worsened distinct features of the disease as shown on both macroscopic and microscopic levels. Both CPs and WTAs in *S. aureus* are clearly important in the pathogenesis of staphylococcal infections (O’riordan and Lee, 2004; Wanner et al., 2017). CPs enhance staphylococcal virulence by impeding phagocytosis, resulting in bacterial persistence in the bloodstream of infected hosts. *S. aureus* CPs also promote abscess formation (O’riordan and Lee, 2004). Moreover, WTA enhances the staphylococcal virulence and the skin abscess induction in an animal model (Wanner et al., 2017). The effects on antibiotic resistance and adhesion could be caused by impaired biosynthesis of WTA and CP due to the absence of LcpC. Our results provide additional evidence for a new mechanism by which CPs and WTAs in *S. aureus* enhance the pathogenesis through enhanced antibiotic resistance and adhesion capability to host cells.

Inhibitors for WTA biosynthesis have been investigated as combination agents to restore β-lactam efficacy against MRSA (Chen et al., 2009; Wang et al., 2013), with promising results. These inhibitors targeted TarG, the WTA transporter protein (Wang et al., 2013). Since impairment of LcpC, a bifunctional ligase of WTAs and CPs to PG, reduced resistance to β-lactam and the pathogenicity of MRSA in the host, these findings from our study suggest that LcpC might be another effective or a better target for drug development against MRSA. Additionally, LCP proteins exist in most Gram-positive pathogens (Hubscher et al., 2008) and they also have cell wall ligase activities in *Streptococcus pneumoniae* (Eberhardt et al., 2012) and *B. anthracis* (Liszewski Zilla et al., 2015). Therefore, LcpC homologs in these pathogens can be potential new targets to combat associated infections.

The work presented herein exemplifies the growing appreciation of the importance of LCP proteins in *S. aureus* and further expands the list of functions for LCP proteins. It is the first time to demonstrate that LcpC contributes to the antimicrobial resistance in *S. aureus* and its pathogenicity development. Our findings might help in the development of new treatment strategies for MRSA-associated infections, even for other multiple-drug-resistant Gram-positive bacterial infections.

## Data Availability Statement

All datasets generated for this study are included in the article/Supplementary Material.

## Ethics Statement

Animal experiments were performed in strict accordance with the national guidelines for the care and use of laboratory animals. The protocol was approved by the recommendations of the Laboratory Animal Center of Fourth Military Medical University (permit no. 20180502).

## Author Contributions

XZ, FL, and ZW designed the experiments and wrote the manuscript. FL, DZ, YZ, and DQ performed the experiments. FL and DZ analyzed the data.

## Conflict of Interest

The authors declare that the research was conducted in the absence of any commercial or financial relationships that could be construed as a potential conflict of interest.
