# Supplementary material for: Impairment of the Cell Wall Ligase, LytR-CpsA-Psr Protein (LcpC), in Methicillin Resistant Staphylococcus aureus Reduces Its Resistance to Antibiotics and Infection in a Mouse Model of Sepsis
Source: Front Microbiol. 2020 Apr 16;11:557. doi: 10.3389/fmicb.2020.00557 (PMC7212477; doi:10.3389/fmicb.2020.00557)
Supplement: Supplementary file 1 [file Data_Sheet_1.DOCX]

**Legends for supplementary images.**

**S1 Fig. Antibiotic susceptibility testing by spot assay for MRSA BA01611 and MRSA Mu50.** Serial dilutions of *S. aureus* were spotted on the MHA+2% NaCl medium, then incubated for 24h at 35°C. The first 1-μL spot dilution corresponds to 10^5^ CFU. **(A)** MRSA BA01611 cultured in the MHA+2% NaCl medium with no antibiotic, 2 mg/L ciprofloxacin, 20 mg/L tetracycline, 1 mg/L chloramphenicol, 70 mg/L streptomycin. **(B)** MRSA Mu50 cultured in the MHA+2% NaCl medium with no antibiotic, 4 mg/L ciprofloxacin, 5 mg/L tetracycline, 1 mg/L chloramphenicol, 50 mg/L streptomycin.

**S2 Fig.** **β-lactam and glycopeptides susceptibility assay by growth curve in Newman and RN4220**. The growth assay of the wild-type, the *lcpC* mutant, and the *lcpC* complementary strains in the TSB medium was carried out at 37°C. **(A)** MSSA Newman in the TSB medium with no antibiotic, 0.5 mg/L oxacillin, 0.3 mg/L cefazolin, 0.15 mg/L penicillin, 2 mg/L teicoplanin, 2 mg/L vancomycin. **(B)** MSSA RN4220 in the TSB medium with no antibiotic, 0.5 mg/L oxacillin,0.2 mg/L cefazolin, 0.1 mg/L penicillin, 1.3 mg/L teicoplanin, 1mg/L vancomycin. Data are represented as the means ± S.D. (error bars) of the results of three independent experiments.

**S3 Fig. Adhesion of *S. aureus* BA01611 , Mu50 and RN4220** **to cell lines by flow cytometry.** The adhesion between *S. aureus* strains (BA01611 WT, BA01611 Δ*lcpC*, BA01611 Δ*lcpC*:: *lcpC*; Mu50 WT, Mu50 Δ*lcpC*, Mu50 Δ*lcpC*:: *lcpC*; Newman WT, Newman Δ*lcpC*, Newman Δ*lcpC*:: *lcpC*; RN4220 WT, RN4220 Δ*lcpC*, RN4220 Δ*lcpC*:: *lcpC*) encoding GFP and four cell lines (A549, MCF-7, HCMEC and HaCaT) were analyzed by flow cytometry at 1-hour post adhesion. Ten thousand host cells were collected and GFP fluorescence intensity was calculated by CytoFLEX S (Beckman Coulter). Count-Fluorescence panels and FSC-Fluorescence panels showed that the GFP fluorescence distribution of cell populations. Red, green and orange represent the host cells adhered by WT strains, Δ*lcpC* strains, and △*lcpC::lcpC* strains, respectively. The mean fluorescence intensity (MFI) of each sample harboring specific cells was calculate using the CytExpert 2.3 (Beckman Coulter) software. Relative MFI = MFI of host cells adhered by *S. aureus* strains / MFI of control host cells. Two-tailed unpaired Student’s *t*-test was performed. NS, not significant, *P<0.05, **P<0.01, ***P<0.001. n = 5 replicates.

**S4 Fig. TEM analysis of *S. aureus*** **at the logarithmic phase.** Analysis of cell morphology on *S. aureus* cultured in the TSB medium during the logarithmic phase. From the left to the right: **(A)** BA01611 WT, BA01611 Δ*lcpC*, BA01611 Δ*lcpC*:: *lcpC* **(B)** Mu50 WT, Mu50 Δ*lcpC*, Mu50 Δ*lcpC*:: *lcpC* **(C)** RN4220 WT, RN4220 Δ*lcpC*, RN4220 Δ*lcpC*:: *lcpC*.

**S5 Fig.** **TEM and SEM analysis of Newman Analysis.** Analysis of cell morphology of Newman cultured in the TSB medium overnight and 1 mL of stationary phase culture was collected for TEM assay and SEM. From the left to the right: **(A)** TEM analysis of Newman WT and its derivatives. **(B)** SEM analysis of Newman WT and its derivatives.
