# Supplementary material for: Impairment of the Cell Wall Ligase, LytR-CpsA-Psr Protein (LcpC), in Methicillin Resistant Staphylococcus aureus Reduces Its Resistance to Antibiotics and Infection in a Mouse Model of Sepsis
Source: Front Microbiol. 2020 Apr 16;11:557. doi: 10.3389/fmicb.2020.00557 (PMC7212477; doi:10.3389/fmicb.2020.00557)
Supplement: Supplementary file 2 [file Table_1.DOCX]

**Table S1 Strains, cell lines and plasmids used in this study**

| Strains or plasmids | Description | Source |
| --- | --- | --- |
| **Strains** | | |
| *S. aureus* BA01611 | Wild type | 25 |
| *S. aureus* BA01611 Δ*lcpC* | Deletion of *lcpC* in BA01611 | This study |
| *S. aureus* BA01611 Δ*lcpC*::*lcpC*  *S. aureus* Mu50 | Mutant complemented with *lcpC*  Wild type | This study |
| *S. aureus* Mu50 Δ*lcpC* | Deletion of *lcpC in* Mu50 | This study |
| *S. aureus* Mu50 Δ*lcpC*::*lcpC* | Mutant complemented with *lcpC* | This study |
| *S. aureus* Newman | Wild type | Laboratory strain |
| *S. aureus* Newman Δ*lcpC* | Deletion of *lcpC* in Newman | This study |
| *S. aureus* Newman Δ*lcpC*::*lcpC* | Mutant complemented with *lcpC* | This study |
| *S. aureus* RN4220 | Wild type | Laboratory strain |
| *S. aureus* RN4220 Δ*lcpC* | Deletion of *lcpC* in RN4220 | This study |
| *S. aureus* RN4220 Δ*lcpC*::*lcpC* | Mutant complemented with *lcpC* | This study |
| *E. coli* DH5α | Cloning host strain | Laboratory strain |
| BA01611 GFP | BA01611 expressed GFP | This study |
| BA01611 Δ*lcpC* GFP | BA01611 Δ*lcpC* expressed GFP | This study |
| BA01611 Δ*lcpC*::*lcpC* GFP | BA01611 Δ*lcpC*::*lcpC* expressed GFP | This study |
| Mu50 GFP | Mu50 expressed GFP | This study |
| Mu50 Δ*lcpC* GFP | Mu50 Δ*lcpC* expressed GFP | This study |
| Mu50 Δ*lcpC*::*lcpC* GFP | Mu50 Δ*lcpC*::*lcpC* expressed GFP | This study |
| Newman GFP | Newman expressed GFP | This study |
| Newman Δ*lcpC* GFP | Newman Δ*lcpC* expressed GFP | This study |
| Newman Δ*lcpC*::*lcpC* GFP | Newman Δ*lcpC*::*lcpC* expressed GFP | This study |
| RN4220 GFP | RN4220 expressed GFP | This study |
| RN4220 Δ*lcpC* GFP | N4220 Δ*lcpC* expressed GFP | This study |
| RN4220 Δ*lcpC*::*lcpC* GFP | RN4220 Δ*lcpC*::*lcpC* expressed GFP | This study |
| **Cell lines** | | |
| A549 | Lung adenocarcinoma cells, ATCC CCL-185 | Obtained from the ATCC |
| MCF-7 | Breast cancer cells, ATCC HTB-22 | Obtained from ATCC |
| HCMEC | Human Cardiac Microvascular Endothelial Cells, ZY-603 | Obtained from Shanghai Ze Ye Biotech Co ., Ltd |
| HaCaT | Human skin keratinocytes, ZY-504 | Obtained from Shanghai Ze Ye Biotech Co ., Ltd |
| RAW 264.7 | Murine macrophage cell line, ATCC TIB-71 | Obtained from ATCC |
| **Plasmids** | | |
| pKZ2-Δ*lcpC* | Knockout vector | This study |
| pKZ2-*lcpC*-*Xho*1 | Complementary plasmid | This study |
| pSB2019 | Expression vector of GFP | 44 |
