# Supplementary material for: Impairment of the Cell Wall Ligase, LytR-CpsA-Psr Protein (LcpC), in Methicillin Resistant Staphylococcus aureus Reduces Its Resistance to Antibiotics and Infection in a Mouse Model of Sepsis
Source: Front Microbiol. 2020 Apr 16;11:557. doi: 10.3389/fmicb.2020.00557 (PMC7212477; doi:10.3389/fmicb.2020.00557)
Supplement: Supplementary file 3 [file Table_2.DOCX]

**Table S2. Primers used in this study**

| **Primer name** | **Oligonucleotide(5’-3’)** | **Apllication** |
| --- | --- | --- |
| up-lcpC-F | TGACGTTGAGCCTCGGAACCGGTACCCAAATAGACAATGTTATTCCGCAAT | *lcpC* deletion & Δ*lcpC* complementation |
| up-lcpC-R | TTTACTTTTAGACGAACGTTTCAATCCC | *lcpC* deletion |
| dw-lcpC-F | AACGTTCGTCTAAAAGTAAATAAACTAAGGGAAAATAAGTTATC | *lcpC* deletion |
| dw-lcpC-R | AGATCTCCGGCGGCCGCTCGGAATTCTACAATACAGCATATGCTTTTCTAAAATTCG | *lcpC* deletion & Δ*lcpC* complementation |
| lcpC-XhoI-F | TCTAGAGTAGCTCGAGTAAACTAAGGGAAAATAAGTTATCAAAATTGTACAC | Δ*lcpC* complementation |
| lcpC-XhoI-R | CCTTAGTTTACTCGAGCTACTCTAGATTATCTTTTAATAACTTAGTACTTTCAG | Δ*lcpC* complementation |
| lcpC-F | TTGGCCGTGGTACTACTGAAA | *lcpC* deletion & Δ*lcpC* complementation |
| lcpC-R | CACCGCCGATGCTATCTATC | *lcpC* deletion & Δ*lcpC* complementation |
| pKZ2-F | AATGTCACTAACCTGCCCCGT | pkZ2 plasmid sequence |
| pKZ2-R | AAAGGGTGTGCTTAAATCGGG | pkZ2 plasmid sequence |
| TNF-α (forward) | CAGGCGGTGCCTATGTCTC | Real-time PCR |
| TNF-α (reverse) | CGATCACCCCGAAGTTCAGTAG | Real-time PCR |
| IL-1β(forward) | GAAATGCCACCTTTTGACAGTG | Real-time PCR |
| IL-1β (reverse) | TGGATGCTCTCATCAGGACAG | Real-time PCR |
| IL-6(forward) | CTGCAAGAGACTTCCATCCAG | Real-time PCR |
| IL-6(reverse) | AGTGGTATAGACAGGTCTGTTGG | Real-time PCR |
| IL-10 (forward) | AGCCTTATCGGAAATGATCCAGT | Real-time PCR |
