# Supplementary figures and images for: Impairment of the Cell Wall Ligase, LytR-CpsA-Psr Protein (LcpC), in Methicillin Resistant Staphylococcus aureus Reduces Its Resistance to Antibiotics and Infection in a Mouse Model of Sepsis
Source: Front Microbiol. 2020 Apr 16;11:557. doi: 10.3389/fmicb.2020.00557 (PMC7212477; doi:10.3389/fmicb.2020.00557)

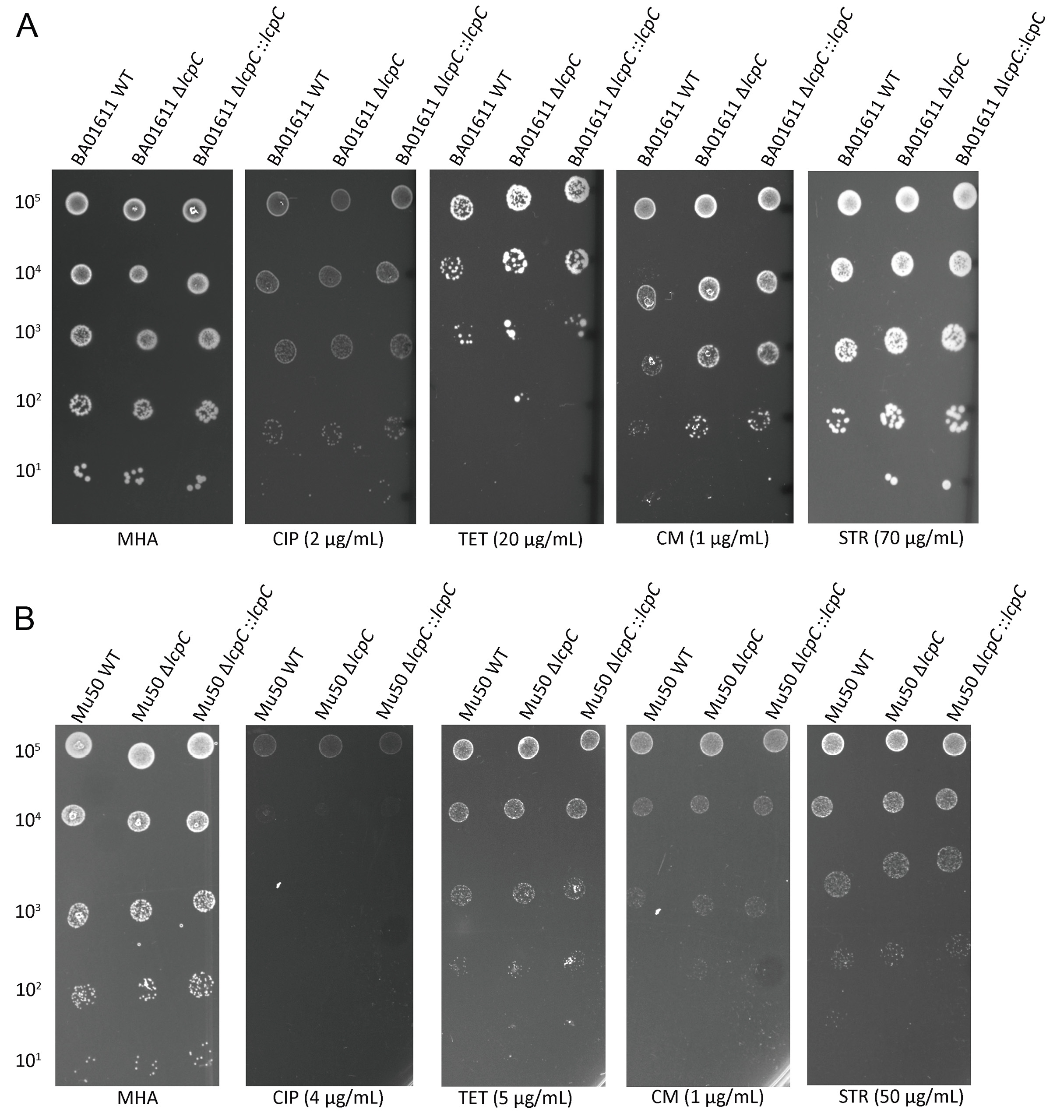

Supplement: Supplementary file 4 [file Image_1.JPEG]

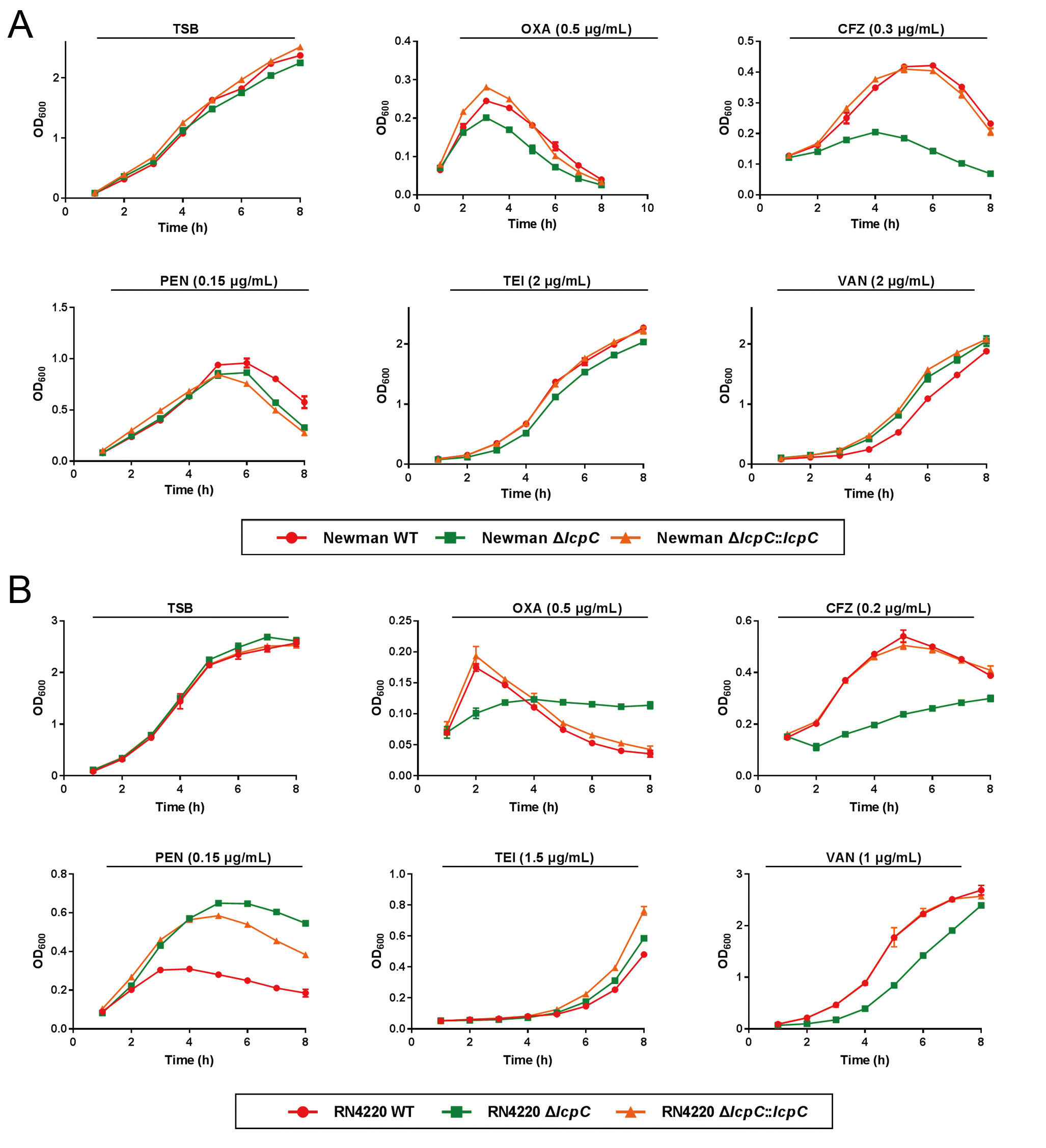

Supplement: Supplementary file 5 [file Image_2.JPEG]

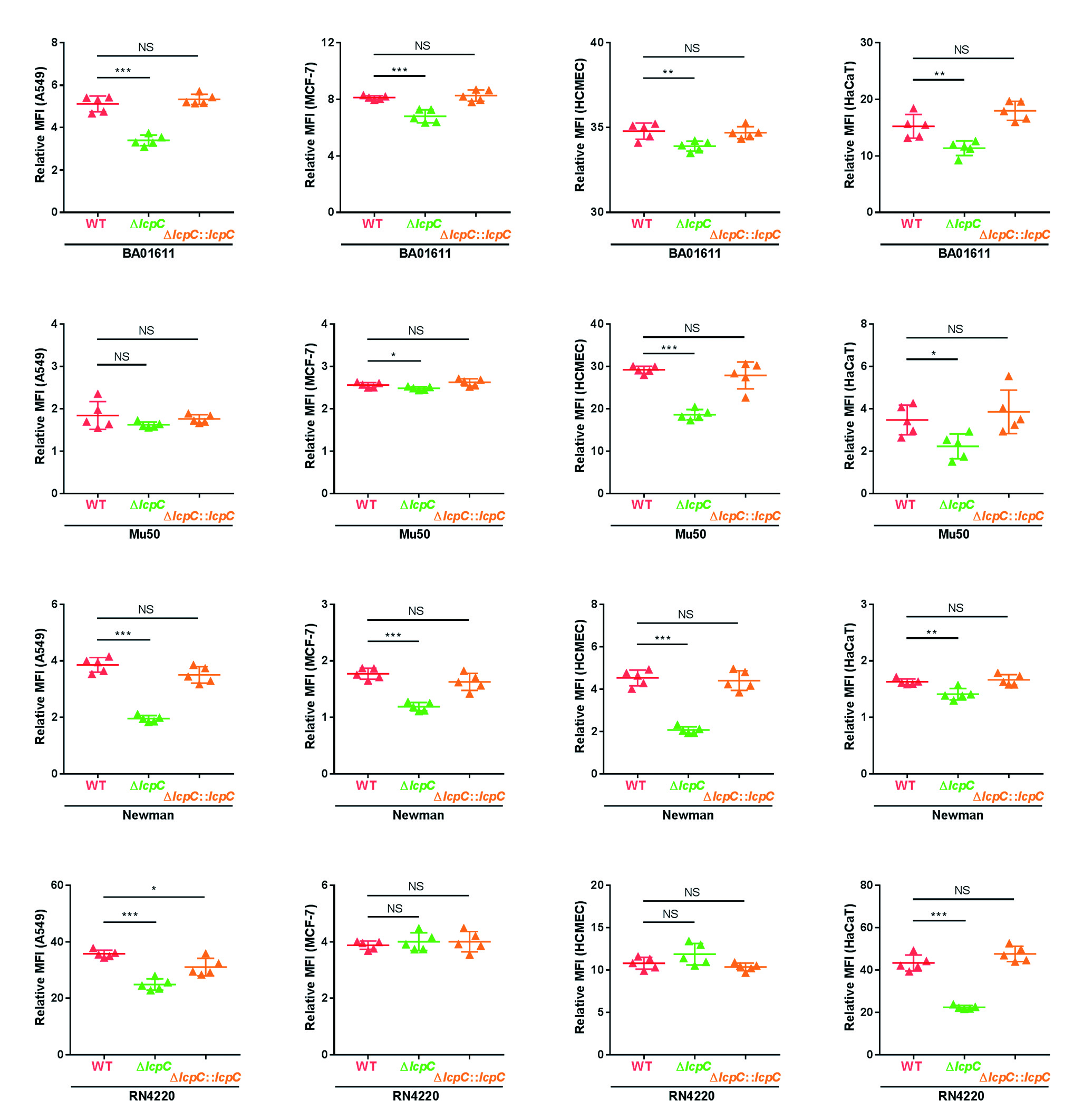

Supplement: Supplementary file 6 [file Image_3.JPEG]

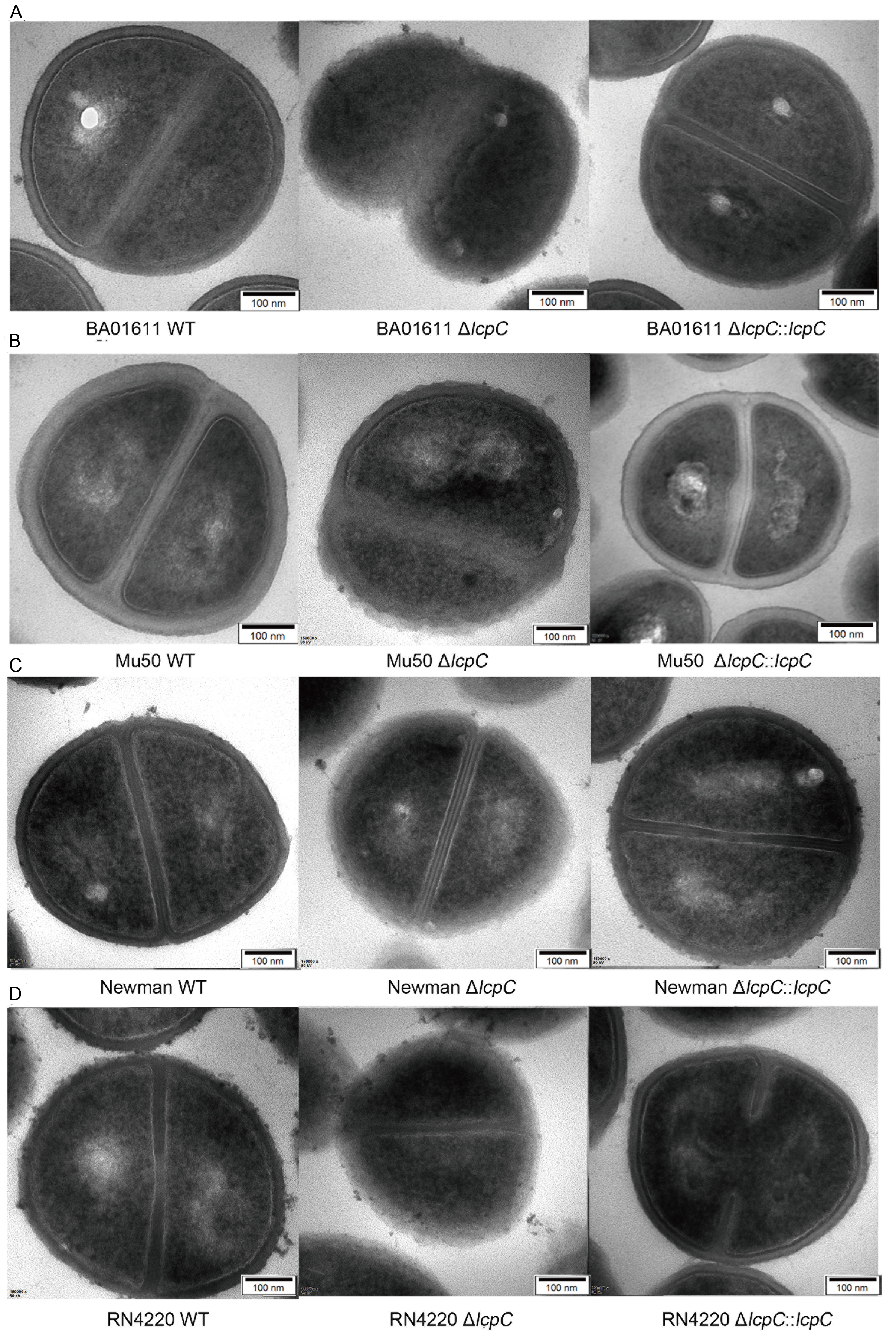

Supplement: Supplementary file 7 [file Image_4.JPEG]

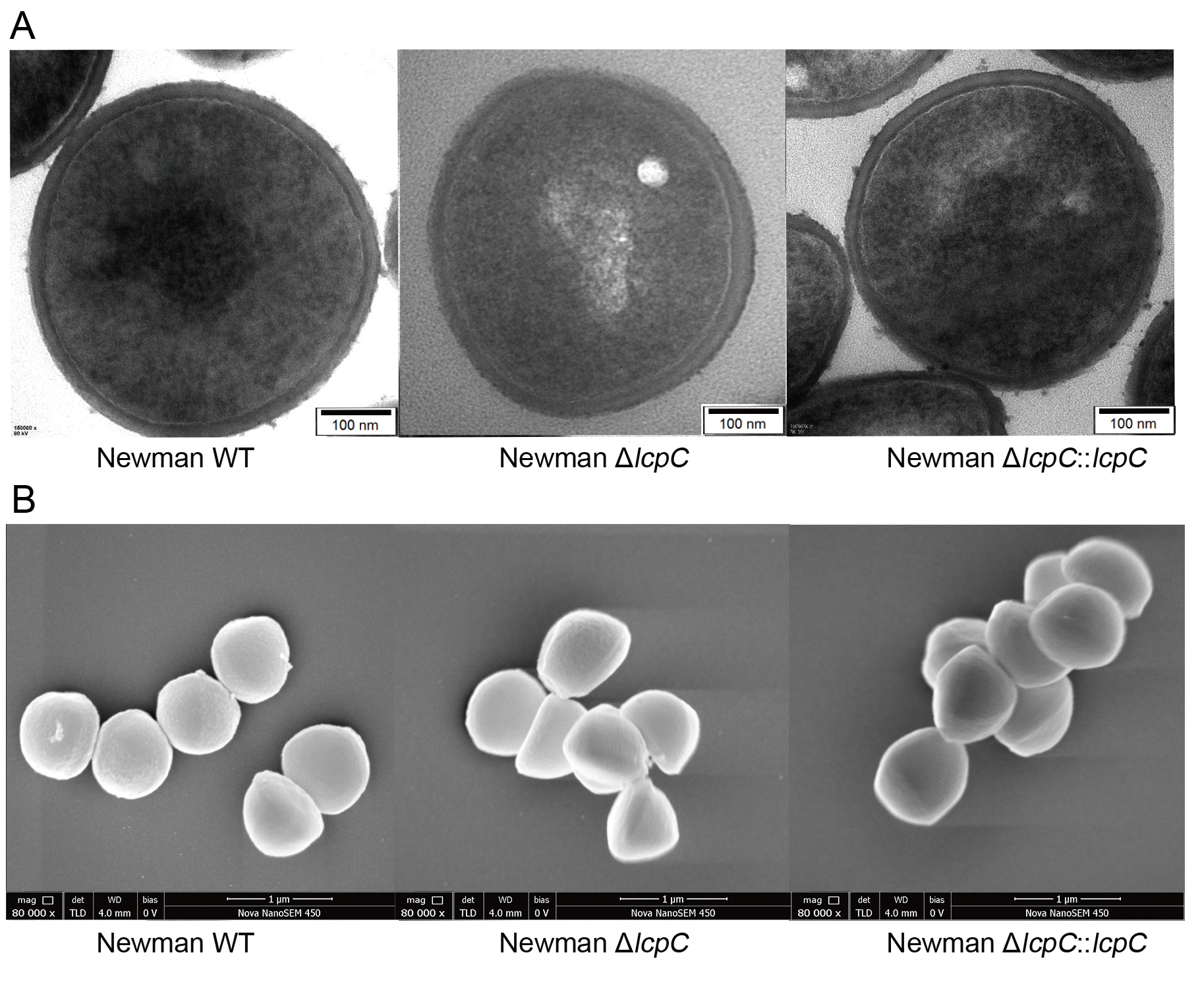

Supplement: Supplementary file 8 [file Image_5.JPEG]
